# *Gfi-1B* Promoter Remains Associated with Active Chromatin Marks Throughout Erythroid Differentiation of Human Primary Progenitor Cells

**DOI:** 10.1002/stem.151

**Published:** 2009-06-11

**Authors:** Benoît Laurent, Voahangy Randrianarison-Huetz, Zahra Kadri, Paul-Henri Roméo, Françoise Porteu, Dominique Duménil

**Affiliations:** a Institut Cochin, Université Paris Descartes, Centre National de la Recherche Scientifique (UMR 8104)Paris, France; b Inserm U567Paris, France; c Service des Thérapies Innovantes, Commissariat à l'Energie AtomiqueFontenay aux Roses, France; d Institut de Radiobiologie Cellulaire et MoléculaireInserm U967/Commissariat à l'Energie Atomique/Paris 7 Fontenay aux Roses, France

**Keywords:** Hematopoietic stem cells, Erythroid differentiation, Regulation of gene expression, Epigenetic processes

## Abstract

Growth Factor Independent-1B (Gfi-1B) is a transcriptional repressor that plays critical roles in the control of erythropoiesis and megakaryopoiesis. Gfi-1B expression was described to be repressed by an autoregulatory feedback control loop. Here, we show that *Gfi-1* transcription is positively regulated early after induction of erythroid differentiation and remains highly active to late erythroblasts. Using chromatin immunoprecipitation assays in CD34^+^ cells from human cord blood, we found that Gfi-1 and GATA-2 in immature progenitors and then Gfi-1B and GATA-1 in erythroblasts are bound to the *Gfi-1B* promoter as well as to the promoter of *c-myc*, a known Gfi-1B target gene. Surprisingly, this Gfi-1/GATA-2–Gfi-1B/GATA-1 switch observed at erythroblast stages is associated to an increase in the *Gfi-1B* transcription whereas it triggers repression of *c-myc* transcription. Accordingly, analysis of chromatin modification patterns shows that HDAC, CoREST, and LSD1 are recruited to the *c-myc* promoter leading to appearance of repressive chromatin marks. In contrast, the *Gfi-1B* promoter remains associated with a transcriptionally active chromatin configuration as highlighted by an increase in histone H3 acetylation and concomitant release of the LSD1 and CoREST corepressors. The repressive function of Gfi-1B therefore depends on the nature of the proteins recruited to the target gene promoters and on chromatin modifications. We conclude that *Gfi-1B* behaves as a lineage-affiliated gene with an open chromatin configuration in multipotent progenitors and sustained activation as cells progress throughout erythroid differentiation.

## INTRODUCTION

The adult hematopoietic system consists of multiple distinct blood cell lineages and is continuously regenerated from hematopoietic stem cells. How cell fate is chosen and maintained during differentiation processes remains elusive. Increasing evidence indicates that coordinated activation of characteristic sets of genes and the silencing of others play an important role in these processes [1]. Recent studies have demonstrated that epigenetic modifications regulate the chromatin state of genes and then their potential to be transcribed. Furthermore, transcription factors can assemble into highly dynamic complexes. In hematopoietic cells, GATA proteins are part of different activator and repressor complexes. A complex between GATA-1 and Gfi-1B was identified on *c-myb* and *c-myc* loci in erythroid cells [2]. These two genes encode for proteins involved in cell proliferation; their repression is requested for the arrest of the cell cycle and the initiation of erythroid differentiation.

Gfi-1 and Gfi-1B are members of the Gfi zinc-finger transcriptional repressor family, whose structure is characterized by an N-terminal repressor domain called SNAG and six C-terminal C2H2 zinc fingers [3]. Gfi-1 and Gfi-1B are differentially expressed in hematopoietic cells. Gfi-1 is expressed in immature progenitors and highly expressed in granulocytes [4,5], whereas Gfi-1B expression in differentiated cells is restricted to erythroid and megakaryocytic cells [6,7]. Analysis of Gfi-1B:green fluorescent protein knockin mice has shown that Gfi-1B expression is dynamically regulated during murine erythropoiesis [6]. Deletion of the *Gfi-1* gene in mouse provokes a severe disturbance of hematopoietic stem cell function due to excessive cycling and severe neutropenia [4,8,9]. *Gfi-1B*-deficient mice are not viable beyond embryonic day 14.5 and fail to produce definitive enucleated red cells [10]. Furthermore, Gfi-1B overexpression in erythroid progenitors strongly disturbs erythroid maturation [7,11]. Gfi-1 and Gfi-1B bind to the same consensus DNA sequence TAAATCAC(A/T)GCA [3,12,13]. Knockin mice in which the *Gfi-1* coding region was replaced by *Gfi-1B* showed that Gfi-1B can replace Gfi-1 in the regulation of hematopoiesis [14].

The mechanisms accounting for the *Gfi-1B* transcriptional regulation are not fully understood. The *Gfi-1B* promoter was cloned and an erythroid-specific promoter region was characterized in K562 cells. Interestingly, while transcriptional activation of the *Gfi-1B* gene relies mainly on GATA proteins, its repression was proposed to be under an autoregulatory feedback loop in NIH3T3 or K562 cells [15,16]. Chromatin regulatory proteins (LSD1, CoREST, and HDAC) have been suggested to mediate transcriptional repression of *Gfi-1B* by Gfi-1B [17]. Evidences for this autoregulation pathway come from studies in NIH3T3 or undifferentiated K562 cells overexpressing Gfi-1B, as well as in MEL or in the spleen and thymus of vav-*Gfi-1B* transgenic mice [18]. This observation is in contradiction with the high levels of Gfi-1B expression observed at the various stages of erythroid differentiation, suggesting that active mechanisms may impede Gfi-1B from repressing its own transcription in differentiating erythroid cells.

We herein study the mechanisms accounting for dynamic regulation of Gfi-1B expression in human multipotent progenitors induced to differentiate toward erythroid lineage. We show that the *Gfi-1B* promoter remains associated with transcriptionally active chromatin modifications during erythroid differentiation despite of the binding of Gfi-1B. Furthermore, as undifferentiated progenitors acquire morphological features of erythroblasts, an increase in the acetylation of histone H3 and a release of the co-repressors, CoREST and LSD1, are observed at the *Gfi-1B* promoter. By contrast, CoREST and LSD1 are recruited together with Gfi-1B at the promoter of a Gfi-1B target gene, *c-myc*, leading to its silencing. Our results thus show that binding of Gfi-1B to its own promoter does not lead to its silencing during erythroid differentiation due release of corepressors.

## MATERIALS AND METHODS

### Cell Cultures

Human UT-7 5.3 cells (a clone of UT-7 cells [19]) were maintained in α-MEM supplemented with 10% heat-inactivated fetal calf serum (FCS), 2 mM L-glutamine, and 5 ng/mL of granulocyte-macrophage colony-stimulating factor. UT-7 5.3 cells were induced to differentiate by addition of 2 U/mL of erythropoietin (EPO); cells synthesizing hemoglobin were detected by benzidine staining. Human K562 cells were maintained in Dulbecco's modified Eagle's medium supplemented with 10% heat-inactivated FCS and 2 mM L-glutamine. K562 cells were induced to differentiate by addition of 0.1 μM of cytosine arabinoside (AraC). Human umbilical cord blood samples were collected from normal full-term deliveries, after informed consent of the mothers according to the approved institutional guidelines of AP-HP (Paris). After isolation of mononuclear cells by density gradient separation, CD34^+^ cells were purified using magnetic bead separation (Stem Cell Technologies, Vancouver, BC, Canada, http://www.stemcell.com). CD34^+^ cells (purity ≥95%) were used immediately or after storage in liquid nitrogen. CD34^+^ cells were maintained for 5 days in serum-free Stem Span medium (Stem Cell Technologies) supplemented with 25 ng/mL of stem cell factor (SCF), 10 ng/mL of interleukin-3 (IL-3), 10^-6^ M of dexamethasone, and 2 U/mL of EPO. Then, cells were induced to differentiate during 5–6 days in Stem Span medium supplemented with 25 ng/mL of SCF and 2 U/mL of EPO. Recombinant human EPO was a gift from Dr. M. Brandt (Roche Diagnostics, Basel, Switzerland, http://www.roche-applied-science.com). Others cytokines were purchased from Promocell Bioscience Alive (Heidelberg, Germany, http://www.promocell.com).

### Cell Transduction

UT-7 5.3 cells were infected with a lentiviral vector carrying a Gfi-1B-specific shRNA, with a sequence previously described [11]. At 48 hours after infection, nuclear proteins and mRNAs were prepared.

### Polymerase Chain Reaction

Oligo(dT)-primed cDNA were synthesized from total RNAs using the superscript II reverse transcriptase (Invitrogen, Carlsbad, CA, http://www.invitrogen.com). Then 2 μL of cDNA were then amplified with Taq polymerase using the following thermal cycling program: 95°C for 5 minutes, 40 cycles of 5 seconds at 95°C, 5 seconds at 60°C, and 10 seconds at 72°C, followed by a 5-minute extension time at 72°C. The primer sequences used were as follows: *Gfi-1B*: 5′-CAGCACTGAGCCCGCCTTGGACTT-3′ (sense), 5′-GTGGGTGGACAGCGTGGACGAGCG-3′ (antisense), *GAPDH*: 5′-TGGGATTTCCATTGATGACAA-3′ (sense), 5′-CCACCCATGGCAAATTCC-3′ (antisense) and *c-myc* 5′-ATGAAAAGGCCCCCAAGGTAGTTAT-3′ (sense), 5′-GCATTTGATCATGCATTTGAAACAA-3′ (antisense).

### Preparation of Nuclear Extracts and Oligonucleotide Pulldown

For nuclear extract preparation, cells were washed once with phosphate-buffered saline (PBS) and incubated for 10 minutes at 4°C in buffer A (10 mM HEPES, pH 7.6, 3 mM MgCl_2_, 10 mM KCl, 5% glycerol, 0.5% NP-40) containing 1 mM Na_2_VO_4_, 20 mM NaF, 1 mM sodium pyrophosphate, 25 mM β-glycerophosphate and proteinase inhibitors (Roche). After centrifugation, nuclear pellets were resuspended in buffer A containing 300 mM KCl. For oligonucleotide pulldown assays, complexes from 10^7^ cell nuclear extracts were precipitated by addition of 1, 2, or 4 μg double-strand biotin-labeled oligonucleotide at 4°C for 1 hour. DNA-protein complexes were then pelleted using streptavidin-agarose beads (Amersham Biosciences, Piscataway, NJ, http://www.gelifesciences.com). Beads were then washed three times with buffer A and resuspended in 1× Laemmli buffer. The biotinylated oligonucleotides used were: Core Gfi-1B 5′ [Biot]–GACACAAATAATCAGATTGAAAATCAGGGAG–3′, Core Gfi-1B mutated 5′ [Biot]–GACACAAATGGTCAGACCGAAGGTCAGGGAG–3′, Gfi-1B consensus 5′ [Biot]–TGCACAGTAAATCACTGCATTGCGGA–3′, Gfi-1B consensus mutated 5′ [Biot]–TGCACAGTAGGTCACTGCATTGCGGA–3′, GATA consensus 5′ [Biot]–GCCTGGGTAGAGATAAGTGCCTG GC–3′, GATA consensus mutated 5′ [Biot]–GCCTGGGTAGAGACCAGTGCCTGGC–3′, c-myc oligo 5′ [Biot]–GGAAGGTATCCAATCCAGATAGCTGTGCA–3′, c-myc oligo mutated 5′ [Biot]–GGAAGGGGTCCGGTCCAGACCGCTGTGCA–3′.

### Western Blot Analysis and Antibodies

Samples were subjected to 10% SDS-polyacrylamide gel electrophoresis and transferred to nitrocellulose membrane (Schleicher and Schuell). Filters were blocked overnight in 5% skimmed milk Tris-buffered saline (TBS) 0.05% Tween 20 and incubated with the appropriate antibody. Membranes were washed four times in TBS-Tween 20 and incubated for 1 hour with the appropriate peroxidase-conjugated secondary antibody. The primary antibodies used were as follows: Gfi-1 N20 (sc-8558, Santa Cruz Biotechnology Inc., Santa Cruz, CA, http://www.scbt.com), GATA-2 H116 (Santa Cruz Biotechnology, sc-9008), GATA-1 N1 (Santa Cruz Biotechnology, sc-266), c-Myc C-33 (Santa Cruz Biotechnology, sc-42), LSD1 (ab-17721, Abcam, Cambridge, U.K., http://www.abcam.com), CoREST (07-455, Upstate, Charlottesville, VA, http://www.upstate.com), and β-actin (A5441, Sigma-Genosys, Cambridge, U.K., http://www.sigmaaldrich.com). Serum against Gfi-1B was prepared in the laboratory. Briefly, the *Gfi-1B* sequence deleted of the SNAG and zinc-finger domains was fused to the Glutathion-S-Transferase and produced in bacteria. Supernatant was purified on glutathion-sepharose and injected into rabbits. The serum was used as polyclonal antibody. The HorseRadish Peroxydase-conjugated secondary antibodies were as follows: anti-rat (Santa Cruz, sc-2006), anti-rabbit (7074, Cell Signaling Technology, Beverly, MA, http://www.cellsignal.com), anti-goat (6165, SouthernBiotech, Birmingham, AL, http://www.southernbiotech.com), and anti-mouse (7076, Cell Signaling Technology).

### Chromatin Immunoprecipitation Assays

Cells were fixed with 1% formaldehyde for 10 minutes at room temperature before termination with 0.125 M glycine. Cells were then lysed in chromatin immunoprecipitation (ChIP) buffer (1% SDS, 10 mM EDTA, and 50 mM Tris-HCl, pH 8.1) and cross-linked chromatin was sonicated to obtain DNA fragments of 300–800 bp. Immunoprecipitations were performed following the Upstate protocol (http://www.upstate.com). Antibodies used were as follows: GATA-1 C20 (Santa Cruz, sc-1233), Ac Lys9/14-H3 (Upstate, 06-599), di-methyl Lys4-H3 (Upstate, 07-030), di-methyl Lys9-H3 (Abcam, ab-1220), tri-methyl Lys9-H3 (Upstate, 07-523), HDAC1 clone 2E10 (Upstate, 05-100). Antibodies against Gfi-1B, Gfi-1, GATA-2, LSD1, and CoREST used for ChIP assays were the same as for Western blot. The corresponding normal rabbit, goat, or mouse immunoglobulins G (IgG, Santa Cruz) were used as control. The immunoprecipitated DNA was used for polymerase chain reaction (PCR) with the following thermal cycling program: 95°C for 5 minutes, 40 cycles of 30 seconds at 95°C, 30 seconds at 60°C, and 45 seconds at 72°C, followed by a 5-minute extension time at 72°C. The *Gfi1B* promoter sequence was amplified with the primers 5′-GAATTCGAAGTCTTGTGTCC-3′ (sense) and 5′-GTGTGTTTTTCCTCTTCTGT-3′ (antisense), the *c-Myc* promoter with the primers 5′-GAAGGTATCCAATCCAGATAGCTGTGC-3′ (sense) and 5′-GAGCGTGGGATGTTAGTGTAGATAGGG-3′ (antisense), the *Pax6* enhancer with the primers 5′-GTCCGGTGCCTTGAACCAT-3′ (sense) and 5′-GCGCAACTACCGCCTCTAAA-3′ (antisense), and the *Gfi-1* promoter with the primers 5′-TCTTGGCTCCAGGGAGAC-3′ (sense) and 5′-GGAGGGTCCTCGGAGC-3′ (antisense). The primers 5′-CCAGTCTAGTGCATGCCTTCTTAA-3′ (sense) and 5′-CAAGCCAGCGACGCAGT-3′ (antisense) amplify the β*2microglobulin* promoter as control.

## RESULTS

### *Gfi-1B* Gene Expression Is Upregulated at the Transcriptional Level During Human Erythroid Differentiation

**Figure 1:**
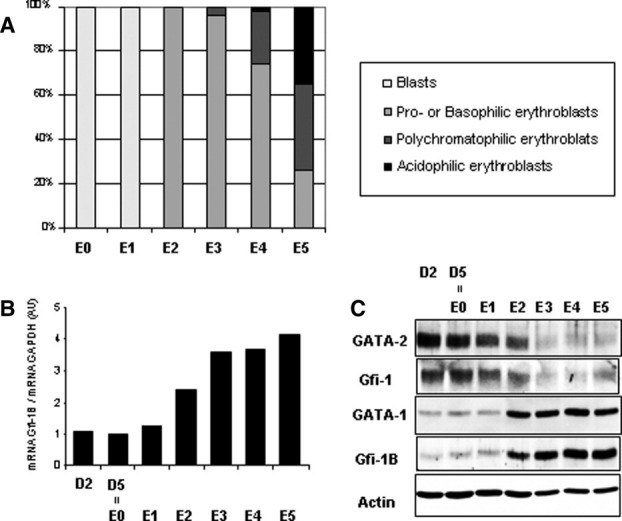
Gfi-1B expression increases after induction of erythroid differentiation and remains elevated along erythroid development. CD34-positive cells were purified from cord blood samples and cultured in a two-phase system. During the first phase of 5 days (D1–D5), CD34^+^ cells were cultured in the presence of interleukin-3, stem cell factor (SCF), erythropoietin (EPO), and dexamethasone. Then, the cells were induced to differentiate in the presence of EPO and SCF (E0–E5). **(A):** Cytology of the cells. Cytospin samples were prepared every day and stained with May-Grunwald-Giemsa. Different subpopulations were characterized under the microscope and the proportion of each subpopulation was evaluated. (**B):** Gfi-1B mRNA expression during erythropoiesis. mRNAs were prepared from CD34^+^ cells before (D2 and D5) and every day after induction of erythroid differentiation (E0–E5). Reverse-transcription polymerase chain reaction was performed using Gfi-1B-specific primers. Data are expressed as the ratio between Gfi-1B and GAPDH mRNA. (**C):** Cell lysates were prepared from the same cell populations as in **(B)**. Total cell extracts were analyzed by SDS-polyacrylamide gel electrophoresis (PAGE) and Western blotting using a Gfi-1B specific antibody. The membrane was stripped and re-hybridized with Gfi-1-, GATA-2- and GATA-1-specific antibodies. Actin was detected by a specific antibody to confirm equal protein loading. These results are representative of three experiments with different samples.

We first studied the kinetics of Gfi-1B mRNA and protein expression during in vitro erythroid differentiation of human CD34 positive cells (CD34^+^). CD34^+^ cells were isolated from cord blood and cells were amplified in a two-phase culture system: a 5-day amplification step (D1–D5) in the presence of SCF, IL-3, EPO, and dexamethasone followed by a 5-day differentiation step (E0–E5) in the presence of SCF and EPO. May-Grunwald-Giemsa (MGG) staining and cytofluorometry analysis demonstrated that the cells remained immature (CD34 positive with no phenotypical changes or morphological features, Fig. 1A and data not shown) during the amplification step (D0 to D5–E0). The expanded cells started to acquire morphologic features of proerythroblasts 2 days after the beginning of the second phase of the culture (E2) and of erythroblasts 3–5 days later (Fig. 1A). During the amplification phase, the expression of Gfi-1B was low at both mRNA and protein levels (Fig. 1B, 1C). Two days after induction of erythroid differentiation (E2), when the cells acquired proerythroblast features, Gfi-1B mRNA level increased 2.5-fold, whereas Gfi-1B protein level increased fourfold. Then a small additional increase in Gfi-1B mRNA and protein levels occurred between days 3 and 4; Gfi-1B expression remained high until E5. We then analyzed the kinetic of expression of erythroid transcription factors known to bind the erythroid specific Gfi-1B promoter in K562 cells [15,16]. It appeared that GATA-1 expression was similar to that of Gfi-1B, with GATA-1 being barely expressed in immature CD34^+^ progenitors and highly expressed in differentiated cells. GATA-2 and Gfi-1 proteins were expressed at high levels in CD34^+^ cells (E0). Their expression decreased early after induction of erythroid differentiation when the cells acquire morphological features of proerythroblasts (E1 and E2; Fig. 1C). Thus, Gfi-1B expression increased when cells acquired erythroid features (E2) and was highly expressed at the end of human erythroid differentiation.

### Binding of GATA-1 and Gfi-1B to the *Gfi-1B* Promoter Increases During Human Erythroid Differentiation

**Figure 2:**
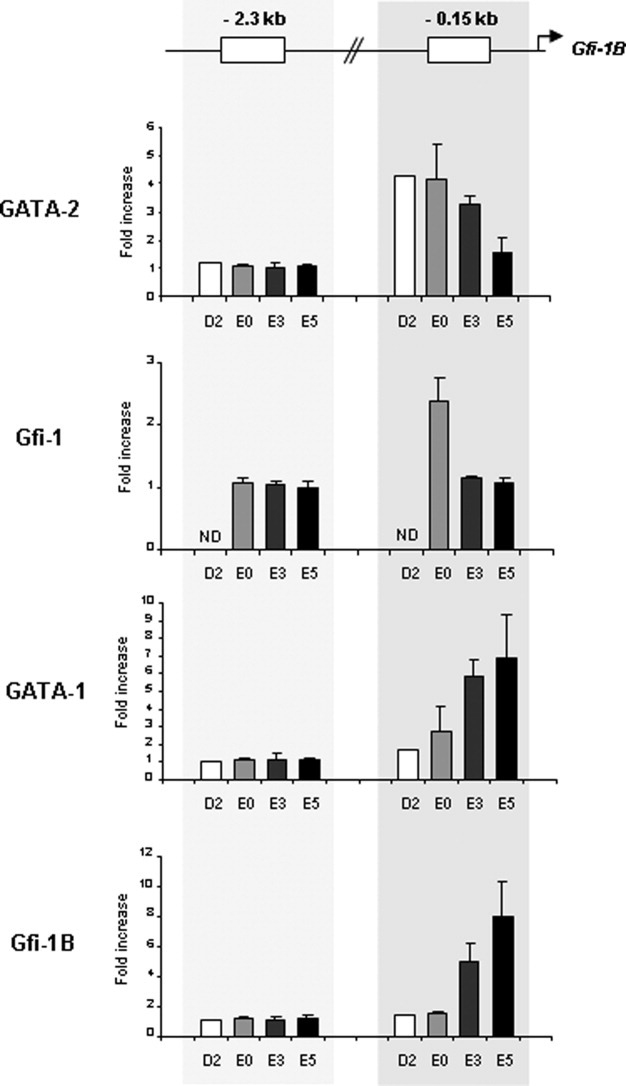
GATA-2 and Gfi-1 and then GATA-1 and Gfi-1B bind at the *Gfi-1B* promoter during erythropoiesis induced from CD34-positive cells. Chromatin immunoprecipitation (ChIP) analyses were performed on CD34^+^ cells (D2 or E0) and 3 or 5 days after induction of erythroid differentiation (E3 and E5) with GATA-2-, GATA-1-, Gfi-1- and Gfi-1B-specific antibodies. Qualitative polymerase chain reaction was performed with two pairs of primers, one that amplifies a sequence located upstream to the *Gfi-1B* promoter (-2.3kb) and the other one that amplifies the *Gfi-1B* promoter (-0.15 kb). Results are expressed as enrichment values (bound/input) relative to immunoprecipitate with IgG antibody and are means ± SD of three independent ChIP experiments (except for the D2 that has been performed only once because of the small number of cells).

To determine when GATA-1, GATA-2, Gfi-1, and Gfi-1B bind to the *Gfi-1B* promoter, ChIP experiments were performed before and after induction of erythroid differentiation using specific antibodies. Immunoprecipitated DNAs were amplified by quantitative real-time PCR with primers surrounding the binding sites of Gfi and GATA sites (-163 to -14) at the *Gfi-1B* promoter (supporting information Fig. 1A). In immature progenitors (D2 and E0), GATA-2 and Gfi-1 were recruited at the Gfi-1B promoter (fold increases of 4.2 and 2.3, respectively), whereas GATA-1 and Gfi-1B were absent. When the cells acquired erythroid features (E3), a decreased recruitment of GATA-2 and Gfi-1 and an increased recruitment of GATA-1 (fold increase 6.0) and Gfi-1B (fold increase 4.4) on the *Gfi-1B* promoter were observed (Fig. 2). GATA-1 and Gfi-1B recruitment was maximal when cells were differentiated (fold increase 7 and 8, respectively, at E5). As control, qualitative PCR was performed with primers located upstream of the *Gfi-1B* promoter on the same immunoprecipitated materials and no specific enrichment was observed. Thus, we conclude that, throughout the erythroid differentiation, the binding of the erythroid transcription factors at the *Gfi-1B* promoter correlate with their expression pattern

### Gfi-1/GATA-2, Gfi-1B/GATA-1 Switch Also Occurs at the *C-Myc* Promoter

**Figure 3:**
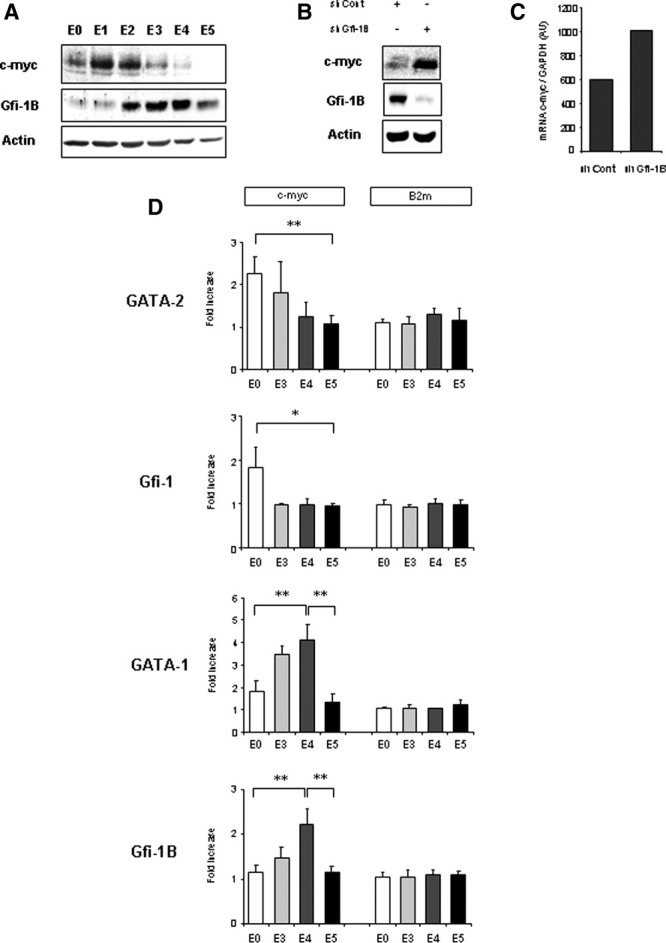
GATA-2 and Gfi-1 then GATA-1 and Gfi-1B bind to *c-myc* promoter during erythroid differentiation. (**A):** The c-myc expression in CD34^+^ cells that are induced to differentiate in the presence of erythropoietin (EPO) and stem cell factor. Cells were harvested and lysed the first day (E0) and then every day (E1–E5) after induction of erythroid differentiation by EPO. Total cell extracts were analyzed by SDS-polyacrylamide gel electrophoresis and Western blotting using Gfi-1B-, c-myc- or actin-specific antibodies. **(B, C):** The c-myc accumulation at the mRNA (**C)** and protein (**B)** levels after of Gfi-1B depletion. UT-7 cells were infected with a lentiviral vector carrying a Gfi-1B-specifc shRNA. At 48 hours after infection, nuclear proteins were harvested and mRNAs were prepared. Proteins were analyzed by Western blot with c-myc-, Gfi-1B-, and actin-specific antibodies. RNAs were reverse transcribed and amplified with primers specific of c-myc or GAPDH, and the ratio c-myc/GAPDH was calculated. (**D):** Chromatin immunoprecipitation analysis using chromatin from CD34^+^ cells (E0) or from cells harvested 3, 4, or 5 days after induction of erythroid differentiation (E3, E4, and E5) were performed with antibodies against GATA-2, GATA-1, Gfi-1, Gfi-1B, and IgG control. Qualitative polymerase chain reaction was performed using primers amplifying the *c-myc* or the β*2-microglobulin* promoter regions. Results are expressed as enrichment values (bound/input) relative to immunoprecipitate with IgG antibody and are means ± SD of three experiments for each antibody. *, *p* < .03, **, *p* < .008

To investigate why Gfi-1B binding to its own promoter at the erythroblast stages does not lead to its transcriptional repression, we analyzed the binding of GATA and Gfi proteins at the *c-myc* promoter, which has been described as a Gfi-1B target gene in MEL cells [2]. In a first step, we confirmed that c-myc expression was downregulated during erythroid differentiation from human CD34^+^ progenitors (Fig. 3A), as already described in MEL cells [20] and K562 [21]. Then, we analyzed whether *c-myc* transcriptional repression was dependent on Gfi-1B protein. Using lentiviruses transducing shRNA against Gfi-1B, we showed that downregulation of Gfi-1B in undifferentiated cells led to an increase in the c-myc mRNA and protein levels (Fig. 3B, 3C), suggesting that Gfi-1B plays a role in the repression of *c-myc* transcription. To analyze the dynamics of GATA and Gfi binding at the *c-myc* promoter, ChIP experiments were performed before (CD34^+^ cells, E0) and after induction of erythroid differentiation (E3–E5) with the same antibodies as in Figure 2B. Qualitative PCR was performed with primers specific of *c-myc* promoter (supporting information Fig. 1B) or β*2*-*microglobulin* promoter as control. Before induction of erythroid differentiation, GATA-2 and Gfi-1 were recruited to the *c-myc* promoter (2-fold increase), GATA-1 was poorly recruited and Gfi-1B was absent. Then, the recruitment of GATA-2 and Gfi-1 decreased and the recruitment of GATA-1 and Gfi-1B increased at the *c-myc* promoter, 3 and 4 days after induction of erythroid differentiation. Thus, as observed for the *Gfi-1B* promoter, the exchange of GATA-2/Gfi-1 for GATA-1/Gfi-1B occurs at the *c-myc* promoter during the erythroid differentiation. However, more importantly, at the end of differentiation (E5) when *c-myc* was no more transcribed, GATA-1 and Gfi-1B were no more found at the *c-myc* promoter (Fig. 3D), whereas GATA-1 and Gfi-1B remained highly bound at the *Gfi-1B* promoter (Fig. 2). Thus, a similar switch between GATA-2/Gfi-1 and GATA-1/ Gfi-1B occurs at the *Gfi-1B* and *c-myc* promoters but an important difference is observed at the end of differentiation (E5): the GATA-1/Gfi-1B complex is released from *c-myc* promoter, whereas it is maintained at the *Gfi-1B* promoter.

### *Gfi-1B* Promoter Remains Associated with Active Chromatin Marks Throughout Erythroid Differentiation

To investigate further the chromatin status at the *Gfi-1B* promoter, during erythroid differentiation, we analyzed the histone post-translational modifications. Histone H3 acetylation on K9 and K14 and/or dimethylation on K4 correlate with transcriptional activation, whereas methylation on K9 is linked to transcriptional repression. The histone post-translational modifications at the *Gfi-1B* promoter were compared with those of *c-myc* promoter by ChIP assays. Cross-linked chromatin from CD34^+^ undifferentiated (E0) or differentiated (E3 and E5) cells was immunoprecipitated with antibodies specific for histone H3 acetylated on lysine 9 and 14 (H3K9/14ac), histone H3 dimethylated on K4 (H3K4me2), or histone H3 dimethylated on lysine 9 (H3K9me2). Promoters of genes transcribed (β2-microglobulin) or silent (*Gfi-1* and *Pax6*) in erythroid cells were used as controls. Interestingly, the *Gfi-1B* promoter exhibited active chromatin marks such as acetylation on K9/K14 (H3-K9/14ac) and methylation on K4 of histone H3 (H3-K4me2) in immature progenitor cells and these marks gradually increased during erythroid differentiation (from E0 to E5). Furthermore, the *Gfi-1B* promoter carried no repressive chromatin marks (di-methylation on K9 of histone H3) throughout erythroid differentiation.

**Figure 4:**
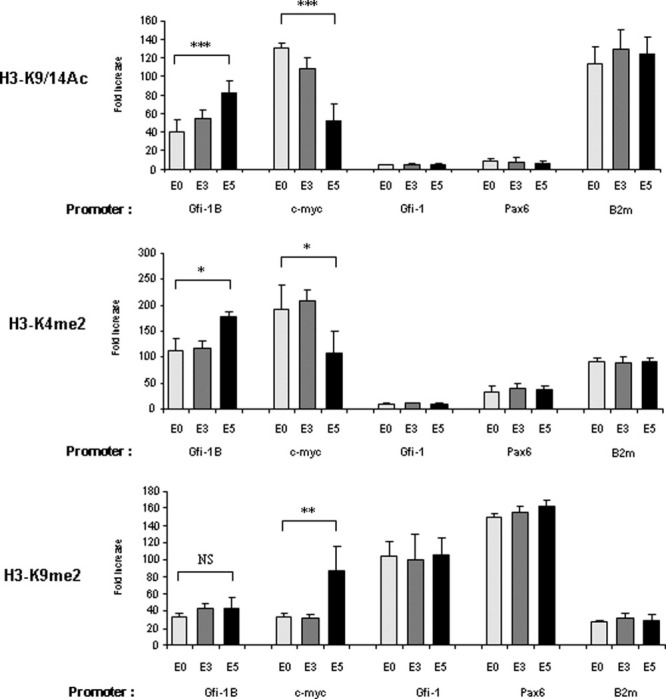
Gfi-1B locus remains permissive for transcription while *c-myc* promoter is silenced in late erythroblasts. Chromatin immunoprecipitation (ChIP) assays using chromatin from CD34^+^ cells harvested the day of induction of erythroid differentiation (E0) and 3 or 5 days later (E3 and E5) were performed with antibodies against acetyl histone H3 (H3-K9/14Ac), antidimethyl histone H3K4 (H3K4me2), antidimethyl H3K9 (H3K9me2), and IgG. Qualitative polymerase chain reaction was performed with primers specific of *Gfi-1B, c-myc, Gfi-1*, and β*2-microglobulin* promoters or *Pax6* enhancer. Results are shown as enrichment values (bound/input) relative to results with IgG antibody and are means ± SD of 2–5 independent ChIP experiments. ***, *p* < .004, **, *p* < .03, *, *p* < .08.

Concerning the *c-myc* promoter, the histone modification patterns evolved differently throughout erythroid differentiation. Active chromatin marks (H3-K9/14ac and H3-K4me2) were present at the *c-myc* promoter in immature progenitors, but these marks decreased during differentiation (E5; Fig. 4). Moreover, the dimethylation on K9 of histone H3, low at E0, was evident at E5 of differentiation at the *c-myc* promoter. The β*2-microglobulin* promoter showed transcriptionally active marks (acetylation on K9 and K14 and methylation on K4 of H3) and no repressive marks (no methylation on H3K9). On the contrary, the *Gfi-1* promoter and the *Pax6* enhancer were associated with repressive histone modifications (no acetylation on K9 or K14, nor methylation on K4 and K9 of histone H3) during the whole process of erythroid differentiation. Thus, our results showed that the *Gfi-1B* promoter remains associated with a combination of active histone modifications, whereas the *c-myc* promoter was associated with negative marks at the end of human erythroid differentiation. These data suggest strongly that the *Gfi-1B* promoter, but not the *c-myc* promoter, stays in a transcriptionally active state throughout erythroid differentiation.

### CoREST and LSD1 Repressors Are Released from the *Gfi-1B* Promoter During Human Erythroid Differentiation

**Figure 5:**
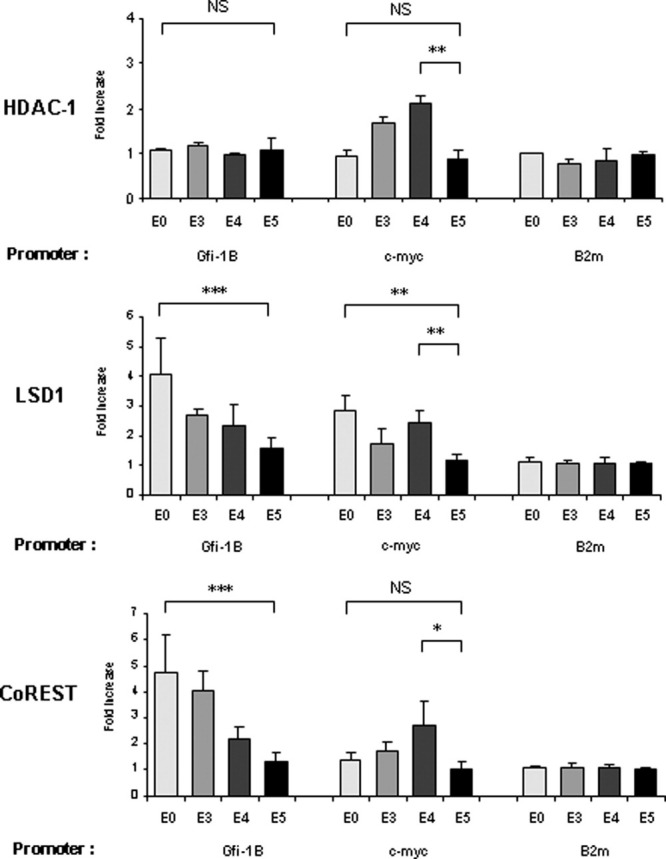
Corepressors are differently recruited at the *Gfi-1B* or at *c-myc* promoters. Chromatin immunoprecipitation analysis with chromatin from CD34^+^ cells harvested the day of the induction of erythroid differentiation (E0) and 3, 4, or 5 days later (E3, E4, and E5) with antibodies against HDAC-1, LSD-1, Co-Rest, and IgG as control. Qualitative polymerase chain reaction were performed with primers specific of *Gfi-1B*, *c-myc*, or the β*2-microglobulin* promoters. Results are shown as enrichment values (bound/input) relative to immunoprecipitation with IgG antibody and are means ± SD of 2–3 independent ChIP experiments. *, *p* < .05, **, *p* < .03, ***, *p* < .01.

Gfi-1 and Gfi-1B have been reported to recruit histone-modifying enzymes, HDAC, LSD1, and CoREST, through their SNAG domain at their target gene promoters [17]. These cofactors are considered to act as corepressors of transcription. HDAC-1 is a histone deacetylase and LSD1 is a lysine-specific demethylase (demethylating K4 of histone H3). CoREST interacts with LSD1 and positively regulates LSD1 function [22]. To determine whether changes in chromatin structure at the *Gfi-1B* or *c-myc* promoters were associated with changes in the recruitment of these enzymes, we analyzed HDAC, LSD1, and CoREST binding in vivo at the *Gfi-1B*, *c-myc*, and β*2-microglobulin* promoters during erythroid differentiation. HDAC1 was not found at the *Gfi-1B* promoter (Fig. 5), which agrees with the increased histone acetylation at the *Gfi-1B* promoter during erythroid development (Fig. 4). Furthermore, LSD1 and CoREST were initially found associated at the *Gfi-1B* promoter before induction of erythroid differentiation and were released from the *Gfi-1B* promoter during erythroid differentiation. By contrast, HDAC1, LSD1 and CoREST are found at the *c-myc* promoter at E3 and E4, when *c-myc* transcription decreased until complete silencing at E5. The binding of this complex at the *c-myc* promoter would be induced specifically by Gfi-1B, as described by others on Gfi-1B target gene promoters [17]. Thus, LSD1 and CoREST cofactors are present at the *Gfi-1B* promoter in CD34^+^ progenitors (E0) but are released at erythroblast stages as histones H3 are hyperacetylated.

### LSD1 and CoREST Bind to c-myc but Not to Gfi-1B Oligonucleotides

**Figure 6:**
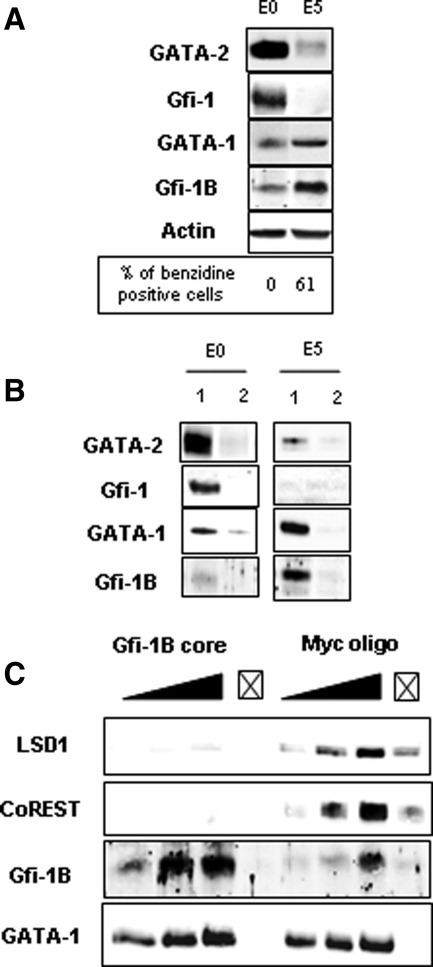
LSD1 and CoREST bind to c-myc but not to Gfi-1B oligonucleotides. (**A):** Gfi and GATA protein expression during erythroid differentiation of UT-7 5.3 cells. UT-7 5.3 cells cultured in the presence of granulocyte-macrophage colony-stimulating factor were induced to differentiate in the presence of erythropoietin (EPO). Cells were lysed the first day (E0) and 5 days (E5) after induction of erythroid differentiation by EPO. The nuclear cell lysates were analyzed by Western blot with Gfi-1, Gfi-1B, GATA-1 and GATA-2 specific antibodies. Actin was detected by a specific antibody to confirm equal protein loading. Cells were stained with benzidine to reveal hemoglobin and the percentage of benzidine positive cells was determined. (**B):** Binding of GATA and Gfi proteins with *Gfi-1B* promoter oligonucleotides before and after erythroid differentiation. DNA affinity precipitation experiment with biotinylated oligonucleotides representing the *Gfi-1B* promoter was performed using nuclear extracts from UT-7 5.3 cells cultured in the presence of GM-CSF (E0) or stimulated by EPO during 5 days (E5). Then 4 μg of wild-type (1) or mutated (2) biotin-labeled oligonucleotides representing the Gfi-1B core of the *Gfi-1B* promoter (-69 to –37 from the start site) were used. The three Gfi-1/Gfi-1B binding sites in the Gfi-1B core oligonucleotide were mutated into GGTC. Proteins bound to the DNA template were analyzed by SDS-polyacrylamide gel electrophoresis (PAGE) and Western blotting using antibodies as indicated. (**C):** Comparison of GATA-1, Gfi-1B, LSD1, and CoREST binding with Gfi-1B and c-myc oligonucleotides. DNA-binding affinity precipitation experiment was performed with biotinylated-oligonucleotides representing *Gfi-1B* (Gfi-1B core as above) or *c-myc* promoter (sequence from -868 to -840 of *c-myc* gene). UT-7 cells were induced to differentiate and lysates were prepared 5 days after induction of differentiation with EPO. Nuclear extracts were incubated with increasing quantities of oligonucleotides (from 1–4 μg indicated by black wedges) of wild-type or with 4 μg of mutated (indicated by a cross) oligonucleotides, precipitated with streptavidin beads and analyzed by Western blotting using antibodies against Gfi-1B or GATA-1, LSD1 and CoREST. This experiment is representative of three experiments.

To determine whether the promoter sequence has a role in the recruitment of activating or repressing cofactors, we carried out in vitro DNA affinity precipitation experiments with nuclear extracts from UT-7 5.3 cells. Indeed, during EPO-induced erythroid differentiation of these cells, GATA and Gfi factors exhibited a similar expression pattern as during the EPO-induced differentiation of CD34^+^ cells (Fig. 6A). As a first step, we verified that we could get the same results by this in vitro technique as by in vivo chromatin immunoprecipitation experiments. Biotinylated oligonucleotides containing the three consensus Gfi-1/Gfi-1B-binding sites (Gfi-1B core) of the *Gfi-1B* promoter were incubated with nuclear extracts from UT-7 5.3 cells before (E0) and after (E5) induction of erythroid differentiation. Before EPO stimulation, GATA-2 and Gfi-1 proteins bound to the Gfi-1B core. Consistent with their expression dropping at later stages of differentiation, the binding of GATA-2 and Gfi-1 decreased in differentiated UT-7 5.3 cells. Conversely, the binding of GATA-1 and Gfi-1B was low at E0 and was elevated on the Gfi-1B core oligonucleotides at late stages of erythroid differentiation (Fig. 6B, E0.1 and E5.1). GATA and Gfi factors did not bind to the oligonucleotides when the three Gfi-1/Gfi-1B binding sites were mutated (Fig. 6B, E0.2 and E5.2). Thus, these results are consistent with in vivo data: (1) a switch between a GATA-2/Gfi-1 and a GATA-1/Gfi-1B occupancy and (2) a sustained binding of GATA-1 and Gfi-1B at late stages of erythroid differentiation. These results also confirm that GATA proteins, GATA-2 then GATA-1 throughout the erythroid differentiation, bind at the consensus Gfi-1/ Gfi-1B binding sites.

Thus, because GATA-1 and Gfi-1B associate with the Gfi-1B core oligonucleotides in vitro, similar oligo pulldown experiments can be carried out to compare molecules bound at the *Gfi-1B* or the *c-myc* promoters. Nuclear extracts from UT-7 5.3 harvested 5 days after induction of erythroid differentiation were incubated with increasing amount of biotinylated oligonucleotides representing either the *Gfi-1B* (Gfi-1B core) or the *c-myc* promoters. The results showed that, at the end of differentiation, a higher amount of Gfi-1B was bound to the Gfi-1B core than to the c-myc oligonucleotides, whereas GATA-1 bound equivalently to the two oligonucleotides. Thus, the LSD1 and CoREST recruitment at the Gfi-1B or *c-myc* promoters is independent of the presence of Gfi-1B but depends on the precise promoter sequence. These in vitro data are consistent with the in vivo data and suggest that the presence of Gfi-1B at the *Gfi-1B* promoter is not sufficient to maintain the binding of LSD1 and CoREST at the end of the erythroid differentiation.

## DISCUSSION

*Gfi-1B*-deficient mice fail to produce definitive enucleated red blood cells [10], suggesting that the maturation of erythroid precursor cells into definitive erythrocytes is dependent on the presence of Gfi-1B. Thus, *Gfi-1B* transcription must be sustained during erythroid differentiation. This is not consistent with Gfi-1B expression being repressed by an autoregulatory feedback control loop, as has been suggested for NIH3T3 or K562 cells [15]. Here, we show that Gfi-1B does not repress its own transcription during erythroid differentiation of human primary CD34^+^ cells. Indeed, the present data show that Gfi-1B expression is maintained at high levels throughout erythroid differentiation in spite of Gfi-1B binding to its own promoter. Luciferase experiments also show that the *Gfi-1B* promoter stays active when transfected into differentiated erythroid cells (data not shown).

Using ChIP assays, we have shown that the transcription of Gfi-1B is dynamically controlled during human erythroid differentiation. Before commitment towards the erythroid lineage (i.e., at the immature progenitor stages), Gfi-1B is barely expressed and GATA-2 and Gfi-1 are bound to its promoter. At the erythroblast stages, GATA-2/Gfi-1 are replaced by GATA-1/Gfi-1B; this switch is associated with an increase in *Gfi-1B* transcription. These results raise the following question: Why is the switch between GATA-2/Gfi-1 and GATA-1/Gfi-1B at the *Gfi-1B* promoter associated with an increase in Gfi-1B transcription? In agreement with results reported by Huang et al. [15], we observed that GATA-2 is a less powerful transcriptional activator than GATA-1 (data not shown). This result may explain why Gfi-1B expression is lower in immature progenitor cells than in differentiated erythroid cells. It has also been proposed that, upon cell maturation, GATA-1 and GATA-2 do not have the same function. For example, GATA-1 displaces GATA-2 at the *Kit* promoter leading to reconfiguration of chromatin organization and modification of *Kit* transcription [23].

Although in overexpression experiments Gfi-1B is able to repress its own transcription (supporting information Fig. 3A, [16]), our results show that while endogenous Gfi-1B together with GATA-1 are found at the *Gfi-1B* promoter during erythroid differentiation, no repression of GATA-mediated *Gfi-1B* transcription is observed. This suggests that the role of Gfi-1B depends on the promoter context. Accordingly, we found that *c-myc*, a known Gfi-1B target gene, is repressed at erythroblast stages. Gfi-1B binds to the proximal *c-myc* promoter and the depletion of Gfi-1B by shGfi-1B in erythroid cells leads to an increase of c-myc mRNA expression, thus demonstrating that *c-myc* silencing does indeed rely on Gfi-1B repressor activity. Therefore, Gfi-1B acts or not as a transcriptional repressor in erythroid cells. It has been suggested that Gfi-1B can exert its repressor activity by interacting with GATA-1 and converting GATA-1-mediated activation to repression at the promoters of *Gfi-1B* and *Bcl*-*x*_*L*_ [16,24] in NIH3T3 cotransfected with GATA-1 and Gfi-1B. However, our results show that, during erythroid differentiation, endogenous Gfi-1B and GATA-1 are bound together at both the *Gfi-1B* and the *c-myc* promoters but this binding leads, in one case, to transcriptional activation and, in the other case, to transcriptional repression.

Furthermore, DNA binding precipitation assays showed that the absence of GATA-1 binding does not impede Gfi-1B recruitment and, vice versa, the absence of Gfi-1B binding does not prevent GATA-1 recruitment at the *Gfi-1B* promoter (supporting information Fig. 2). This provides evidence that Gfi-1B does not need GATA-1 to bind DNA. Our results therefore indicate that GATA-1 and Gfi-1B interaction on a promoter is not sufficient to induce transcriptional repression. The difference in the function of Gfi-1B at *c-myc* and *Gfi-1B* promoters may rather result from the difference in the promoter sequence itself as demonstrated by oligo pulldown experiments. Indeed, at the *Gfi-1B* promoter the “Gfi-1B core” contains three putative Gfi-1/Gfi-1B binding sites that bind both Gfi and GATA proteins, while the proximal *c-myc* promoter contains two GATA and one Gfi-1/Gfi-1B binding sites. This difference could lead to different affinity of GATA and Gfi proteins for the *Gfi-1B* and the *c-myc* promoter sequences; the amount of bound factors may be responsible for recruitment or stability of cofactor complexes at these sites and thereby to different transcriptional activities.

The process of gene repression during development often involves changes in chromatin structure and histone modifications that mimic heterochromatin [25]. Recently, it has been proposed that Gfi proteins could participate in a multistep process that leads to the recruitment of the chromatin regulatory proteins, HDAC, CoREST, and LSD1 followed by heterochromatization and gene silencing [17]. However, our data show that this does not apply to the *Gfi-1B* promoter at erythroblast stages. Indeed, while Gfi-1 (in immature progenitors) or Gfi-1B (in differentiated cells) are bound to the *Gfi-1B* promoter, active chromatin marks are maintained at this promoter from multipotent progenitors to differentiated cells. These active chromatin marks, acetylation and methylation on K4 of histone H3, increase at the *Gfi-1B* promoter during erythroid development. These results were obtained in differentiated erythroid cells from human CD34 immature progenitors. The same active marks were also found at the *Gfi-1B* promoter in K562 cells induced to differentiate in the presence of AraC (supporting information Fig. 3B–E). Our results show that this increase in histone H3 acetylation is due to the lack of HDAC recruitment at the *Gfi-1B* promoter throughout the erythroid differentiation. Histone H3 hyperacetylation due to the absence of HDAC would then lead to the subsequent release of LSD1 and CoREST corepressors. These findings are in agreement with published results indicating that an hypoacetylated histones are better substrates for the LSD1/CoREST complex than hyperacetylated histones [22]. By contrast, HDAC is recruited to the *c-myc* promoter, at erythroblast stages, leading to histone H3 hypoacetylation, recruitment of LSD1 and CoREST, and *c-myc* transcription repression.

The reasons why HDAC is recruited to the *c-myc* but not to the *Gfi-1B* promoter are not clear at this stage. The amount of GATA-1 and/or Gfi-1B bound to the promoter sequence might determine HDAC recruitment and/or stability of the complexes [26]. Indeed, DNA precipitation experiments with oligonucleotides corresponding to the *Gfi-1B* or *c-myc* promoters indicate that although the same quantity of GATA-1 bind to both oligonucleotides, less Gfi-1B is found on the *c-myc* promoter sequence as compared to that of *Gfi-1B*. Equivalent results were obtained by ChIP experiments. Alternatively, additional factors recruited to the *c-myc* and *Gfi-1B* promoters might induce or not HDAC recruitment. Several examples of such cooperation between nuclear proteins in chromatin remodeling process have been described. For example, the co-factor Fog is required at the *Kit* promoter for induction of transition in chromatin conformations by the switch GATA-2/GATA-1 and subsequent inhibition of transcription [23], BHC80 is necessary for the association of LSD1 with unmethylated K4 of H3 histone [27], and ZNF198 stabilizes the LSD1/CoREST/HDAC complex on chromatin [28].

Accumulating evidences indicate that when a stem cell is committed to differentiate towards a specific lineage, global genome reprogramming involves both repression of nonaffiliated genes and selective activation of genes involved in this lineage. The *Gfi-1B* gene follows an epigenetic program corresponding to an erythroid-affiliated gene [29]: in immature progenitors, *Gfi-1B* promoter exhibits active chromatin marks such as dimethylation on K4 of histone H3; *Gfi-1B* locus is thus in a pre-activated state. Then, *Gfi-1B* transcription is maintained throughout erythroid differentiation, these epigenetic marks remaining on its promoter.

In conclusion, we show here that *Gfi-1B* is preactivated in immature progenitors and then activated throughout erythroid differentiation. The loss of the repressive function of Gfi-1B on its own promoter results from the absence of HDAC recruitment, the hyperacetylation of histone H3, and subsequent release of chromatin modifying enzymes responsible for stable silencing. This difference in the HDAC recruitment could be at the origin of the difference in the regulation of the transcription of *Gfi-1B* and of its target genes.
