# Supplementary figures and images for: Gfi-1B Promoter Remains Associated with Active Chromatin Marks Throughout Erythroid Differentiation of Human Primary Progenitor Cells
Source: Stem Cells. 2009 Jun 11;27(9):2153–62. doi: 10.1002/stem.151 (PMC2962905; doi:10.1002/stem.151)

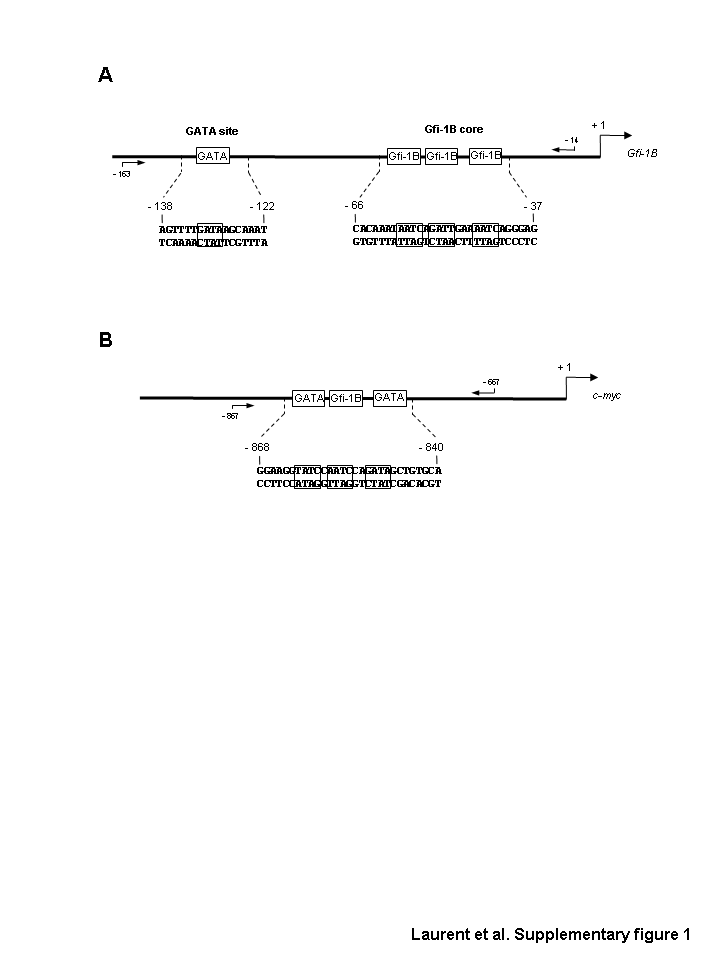

Supplement: Supplementary file 1 [file stem0027-2153-SD1.tif]

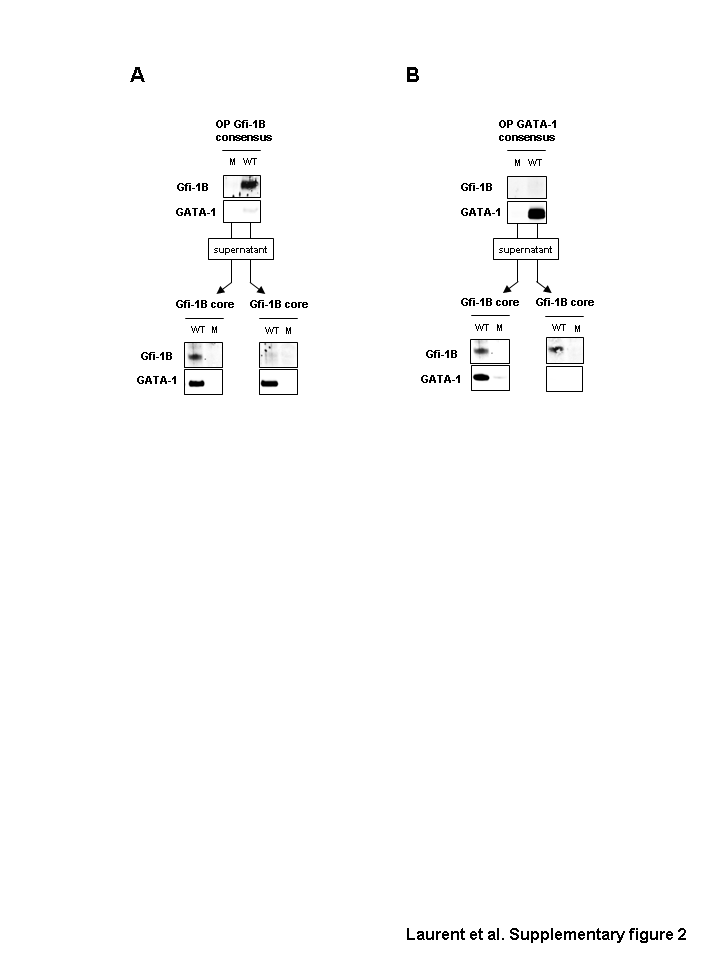

Supplement: Supplementary file 2 [file stem0027-2153-SD2.tif]

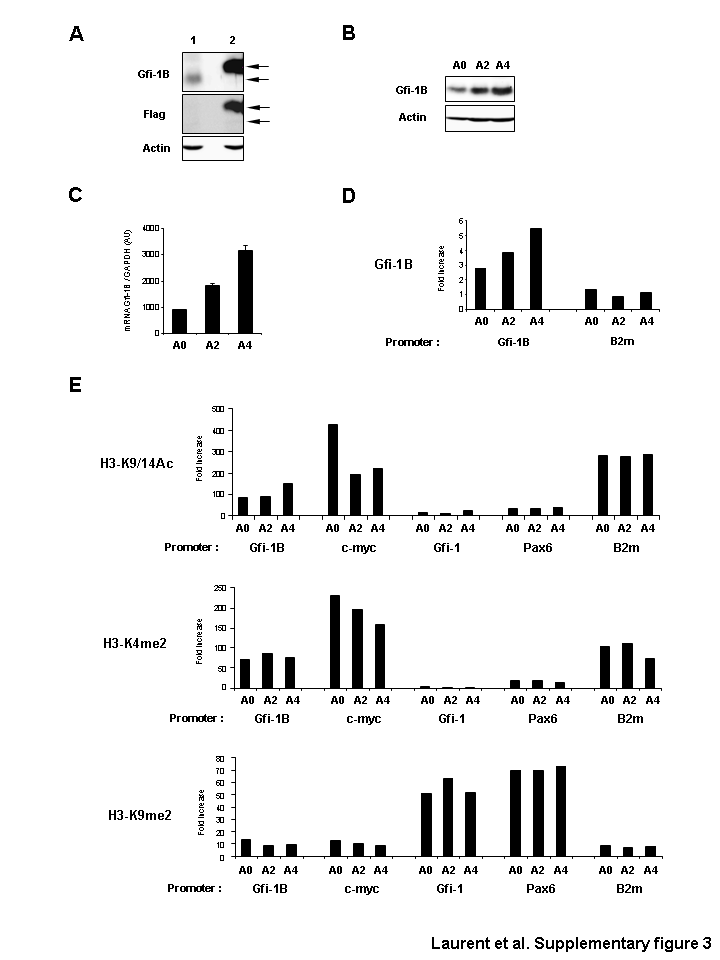

Supplement: Supplementary file 3 [file stem0027-2153-SD3.tif]
